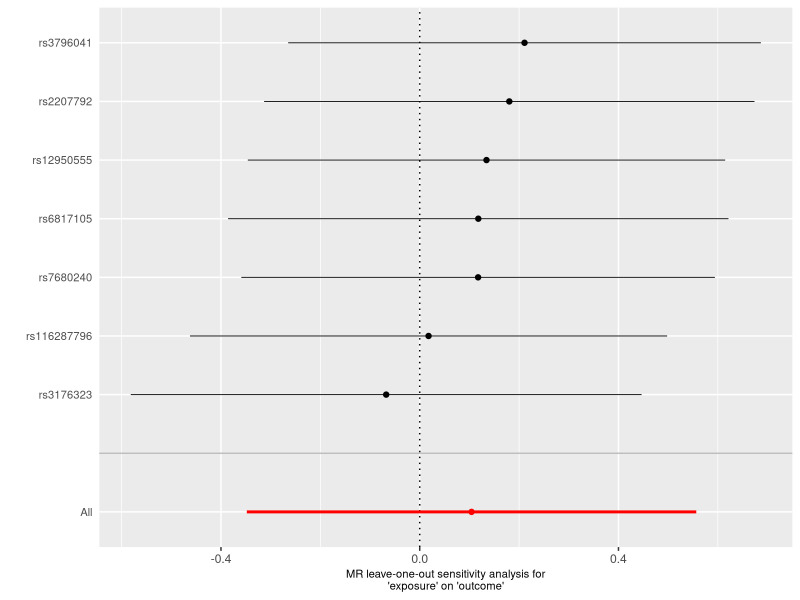# Associations between varicose veins and heart failure: A genetic correlation and mendelian randomization study

**DOI:** 10.1097/MD.0000000000038175

**Published:** 2024-05-17

**Authors:** Ping Guo, Qin Fang, Yan Wang

**Affiliations:** a Division of Cardiology and Department of Internal Medicine, Tongji Hospital, Tongji Medical College, Huazhong University of Science and Technology, Wuhan, China; b Hubei Key Laboratory of Genetics and Molecular Mechanisms of Cardiological Disorders, Wuhan, China.

**Keywords:** GWAS, Heart failure, Mendelian randomization, Varicose veins

## Abstract

Varicose veins and heart failure (HF) are increasingly prevalent. Although numbers of observational studies have indicated that varicose veins might contribute to the risk of HF, the causal relationship between them remains unclear due to the uncontrolled confounding factors and reverse causation bias. Therefore, this study aimed to explore the potential causal relationship between varicose veins and HF. Based on publicly released genome-wide association studies (GWAS), gene correlation was assessed using linkage disequilibrium score (LDSC) regression, and we conducted a two-sample Mendelian randomization (TSMR) analysis to infer the causal relationship. We performed the Inverse variance weighted (IVW) method as the primary analysis, and used Weighted median, MR-Egger, weighted mode, simple mode, and MR-pleiotropy residual sum and outlier (MR-PRESSO) methods to detect and correct for horizontal pleiotropy. LDSC revealed there was a positive genetic correlation between varicose veins and HF (r_g_ = 0.1726184, Se = 0.04511803, *P* = .0001). The results of the IVW method indicated that genetically predicted varicose veins were associated with an increased risk of HF (odds ratio (OR) = 1.03; 95% confidence interval (CI): 1.01–1.06; *P* = .009). Our findings illustrated the significant causal effect of varicose veins on HF, suggesting that people with varicose veins might have a higher risk of HF. The results provided a novel and important perspective into the development mechanism of HF.

## 1. Introduction

Heart failure (HF) is a prevalent chronic disease caused by the progression of various heart diseases to severe stages and the incidence and prevalence of HF continue to rise.^[1]^ Despite apparent improvements in the prevention, epidemiology and management of cardiovascular diseases,^[2]^ there is increasing evidence that HF remains a major healthcare issue. Development of new-onset HF significantly increases the risk for hospitalization and mortality compared with age-matched controls without HF.^[3]^ Indeed, a consistent finding is that HF reduces the quality of life more than other chronic illnesses. Due to its increasing prevalence, an unmet need in public health is to find novel strategies capable of slowing disease progression and reducing its high rate of mortality.^[4]^

Varicose veins, as part of the spectrum of chronic venous disease, occur in the lower limbs due to upright position and hydrostatic pressure. In the United States, approximately 23% of adults have varicose veins.^[5]^ An estimated 22 million women and 11 million men between the ages of 40 to 80 years have varicose veins.^[5]^ Previous studies indicate that 70% to 80% of patients with varicose veins have a family history of the disease.^[6,7]^

Several studies have reported an increased risk of HF in patients with varicose veins, which significantly increases the morbidity and mortality of these patients. For instance, the first follow-up study was Framingham Study on varicose veins and HF.^[8]^ Subsequent cross-sectional cohort studies on the association between varicose veins and HF^[9–13]^ found that patients with varicose veins had a higher prevalence of HF those without varicose veins after age and sex adjustments, and a significantly increased risk of mortality.

Although previous studies have explored their relationship, it is important to note that most of them are observational studies, which have uncontrolled confounding factors and reverse causation bias. Therefore, the exact causal association between varicose veins and HF is currently unknown. However, considering the high prevalence, disability and mortality of heart failure, it is worthwhile to investigate the causal relationship between varicose veins and HF, and thus reducing the substantial disease burden.

In practice, Mendelian randomization (MR) has been widely applied to assess the potential causal relationships between various exposures and clinical outcomes. Compared with traditional observational studies, MR analysis can overcome reverse causation bias, since allelic randomization always precedes the onset of disease. Therefore, the MR approach is conceptually similar to a randomized controlled trial (RCT) but is more widely used and cost-effective. Moreover, random segregation and the independent assortment of genetic polymorphisms at conception enables the MR analysis to minimize the effect of confounding factors by introducing genetic markers as instrument variants (IVs) of exposures. The availability of large-scale genome-wide association studies (GWAS) enables the exploration of causality. Therefore, by applying MR analysis, we aim to assess the causal association between varicose veins and HF by using two-sample Mendelian randomization (TSMR).

## 2. Materials and methods

### 2.1. Study design

**Figure 1.**
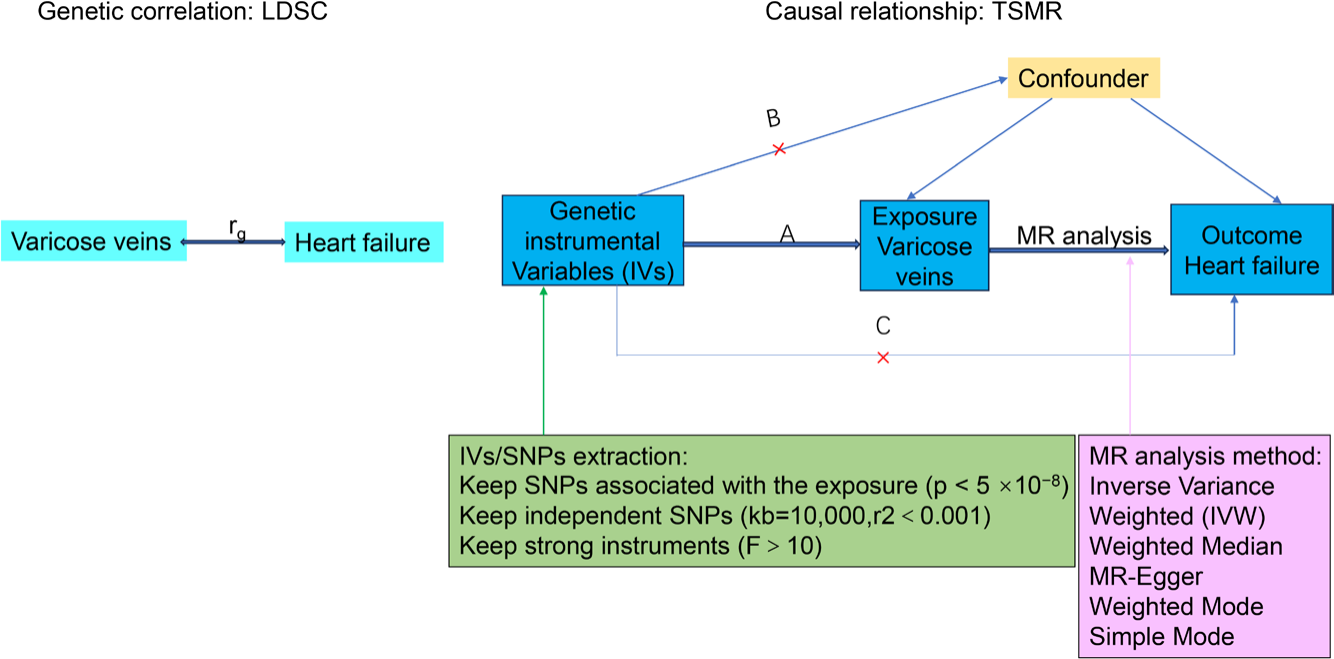
Schematic design of gene correlation and causal relationship between varicose veins and HF. Three key assumptions of the MR study. (A) SNPs are strongly associated with varicose veins; (B) SNPs are independent of confounders; (C) SNPs must only affect HF via varicose veins. HF = heart failure, LDSC = linkage disequilibrium score, MR = Mendelian randomization, SNP = single-nucleotide polymorphism, TSMR = two-simple MR.

A linkage disequilibrium score (LDSC) and TSMR were initially conducted to investigate the genetic correlation and bidirectional causal relationship between varicose veins and HF, respectively. The TSMR analysis was the validated using an independent dataset and employing different MR methods with varying model assumptions. Figure 1 illustrated the schematic design of the study on varicose veins and HF. The IVs used for causal inference in the MR analysis were required to satisfy 3 fundamental assumptions^[14]^: Firstly, the correlation hypothesis, indicating that instruments are strongly associated with the exposure; Secondly, the independence hypothesis: stipulating that instruments are not associated with any other confounders that may be linked to both exposure and outcome; and thirdly, the exclusivity hypothesis, stating that instruments must have no effects on the outcome other than through the exposure (no horizontal pleiotropy exists).

### 2.2. GWAS data sources

The TSMR analysis utilized published summary-level data from GWASs of the relevant traits in predominantly European cohorts, encompassing both males and females. Genetic variants for varicose veins were sourced from the FinnGen consortium R10 release data (finngen_R10_I9_VARICVE, 31,719 cases and 357,111 controls). Summary statistics for HF were derived from the largest available genome-wide meta-analysis of previous HF studies conducted by the HERMES Consortium (ShahS_31919418, 115,150 cases and 1,550,331 controls) (Table 1).

**Table 1:** Detailed information for the GWAS data of varicose veins and heart failure.

Trait	GWAS ID	Ancestry	Sample Size	Case/control	PMID
Varicose veins	finngen_R10_I9_VARICVE	European	388,830	31,719/357,111	NA
Heart failure	ShahS_31919418	European	1,265,481	115,150/1,550,331	36376295

GWAS = genome-wide association studies.

For validation, summary statistics of HF from UK biobank were extracted to assess the consistency of the findings across different datasets (Table S1, Supplemental Digital Content, http://links.lww.com/MD/M504). Additionally, for reverse MR analysis, summary statistics of HF and varicose veins were extracted from the FinnGen consortium R10 release data and UK biobank, respectively (Table S2, Supplemental Digital Content, http://links.lww.com/MD/M505).

### 2.3. Genetic correlation analysis

Genetic correlation (*r*_g_) represents the association of genetic effects between 2 traits that are not affected by environmental factors. Based on GWAS aggregated data, LDSC was used to evaluate genetic correlation analysis of complex phenotypes and to understand the genetic structure of varicose veins and HF. The genetic correlation estimates (*r*_g_) range from −1 to +1, with −1 indicating a perfect negative correlation and +1 indicating a perfect positive correlation.

### 2.4. IVs

In the MR framework, independent instrumental single nucleotide polymorphisms (SNPs) were used as IVs for the exposure (varicose veins) to estimate and test the causal effect on the outcome (HF). The selection of IVs followed specific criteria^[15]^: SNPs robustly associated with each trait (*P* < 5 × 10^−8^); evaluation of independent SNPs according to pairwise linkage disequilibrium (*r*^2^ < 0.001, kb = 10,000). Calculation of the F-statistic to validate the strength of individual SNPs, with a threshold set at F > 10 to indicate no significant weak instrumental bias. The strength of IVs was assessed by calculating the F-statistic using the formula F = *R*^2^ × (*N* − *K* − 1)/*K* × (1 − *R*^2^), where *R*^2^ represents the proportion of variance in the exposure explained by the genetic variants, *N* represents sample size, and *K* represents the number of instruments.

### 2.5. MR analysis

The primary analysis utilized an inverse variance weighted method under a random-effects model to estimate the causal effect.^[16]^ To evaluate horizontal pleiotropy, modified MR-PRESSO was employed to eliminate potential outlier SNPs.^[17]^ When up to 50% of genetic variants are invalid, the total weight of the instrument is derived from the median of the weighted ratio estimates of valid variants, denoting the weighted median method. In order to estimate directional pleiotropic effects, the MR-Egger method and funnel plots were utilized.^[18]^ Furthermore, mode-based methods including simple mode and weighted mode were incorporated to estimate the causal effect of individual SNPs, forming clusters.^[19]^ It was required that the directions of all 5 methods be consistent, and findings with *P* values less than .05 were considered significant.

Heterogeneity among selected IVs was quantified using Cochran’s Q statistics and a leave-one-out cross-validation analysis, aiming to achieve better consistency and higher reliability of the results due to the adoption of different methods for result comparison.^[20,21]^ A reverse causation analysis was performed to exclude the possibility that HF causally affected varicose veins using HF-associated SNPs as IVs. The methods and settings adopted were consistent with those of a forward MR analysis.

### 2.6. Statistical analysis

Statistical analyses were conducted using LDSC software and “TwoSampleMR” package in Rstudio, and the MR estimates were presented as OR with 95% CI. A statistical significance was defined by a *P* value of less than .05.

## 3. Results

### 3.1. Genetic correlation analysis

The results of the genetic correlation analysis are shown in Table 2 and Table S3, Supplemental Digital Content, http://links.lww.com/MD/M506. In general, LDSC analysis showed there was a positive genetic correlation between varicose veins and HF (*r*_g_ = 0.1726184, Se = 0.04511803, *P* = .0001), suggesting a shared genetic basis underlying these 2 complex phenotypes.

**Table 2:** The genetic correlations between varicose veins and heart failure.

Trait1	Trait2	*r*_g_	*r*_g__se	*r*_g__p
Varicose veins	Heart failure	0.1726184	0.04511803	0.0001302801

### 3.2. SNP selection and validation

A total of 86 related SNPs were selected as IVs for varicose veins following specific criteria. The details of the selected IVs were shown in Table S4, Supplemental Digital Content, http://links.lww.com/MD/M507.

### 3.3. Univariable analysis

**Figure 2.**
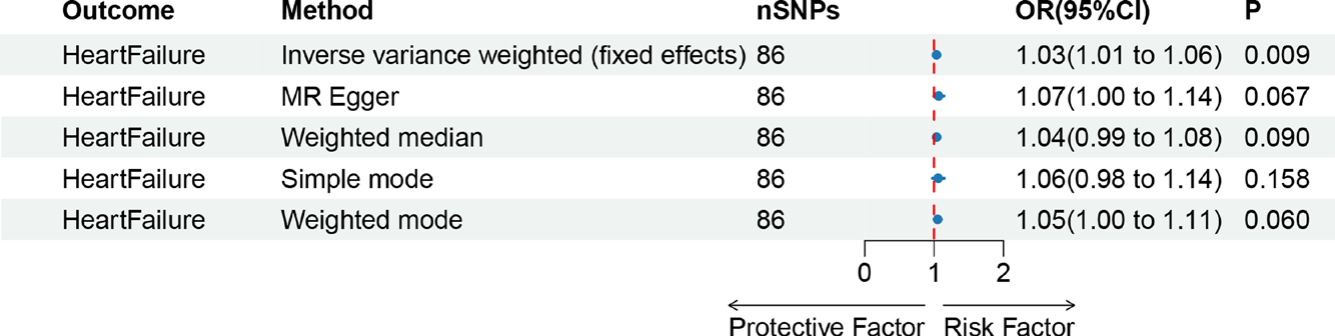
A forest plot to show the odds ratios (ORs) and 95% confidence intervals (CIs) for the effect of varicose veins on heart failure by 5 methods. CI = confidence interval, IVW = inverse-variance weighted, MR = Mendelian randomization, OR = odds ratio, SNP = single-nucleotide polymorphism.

**Figure 3.**
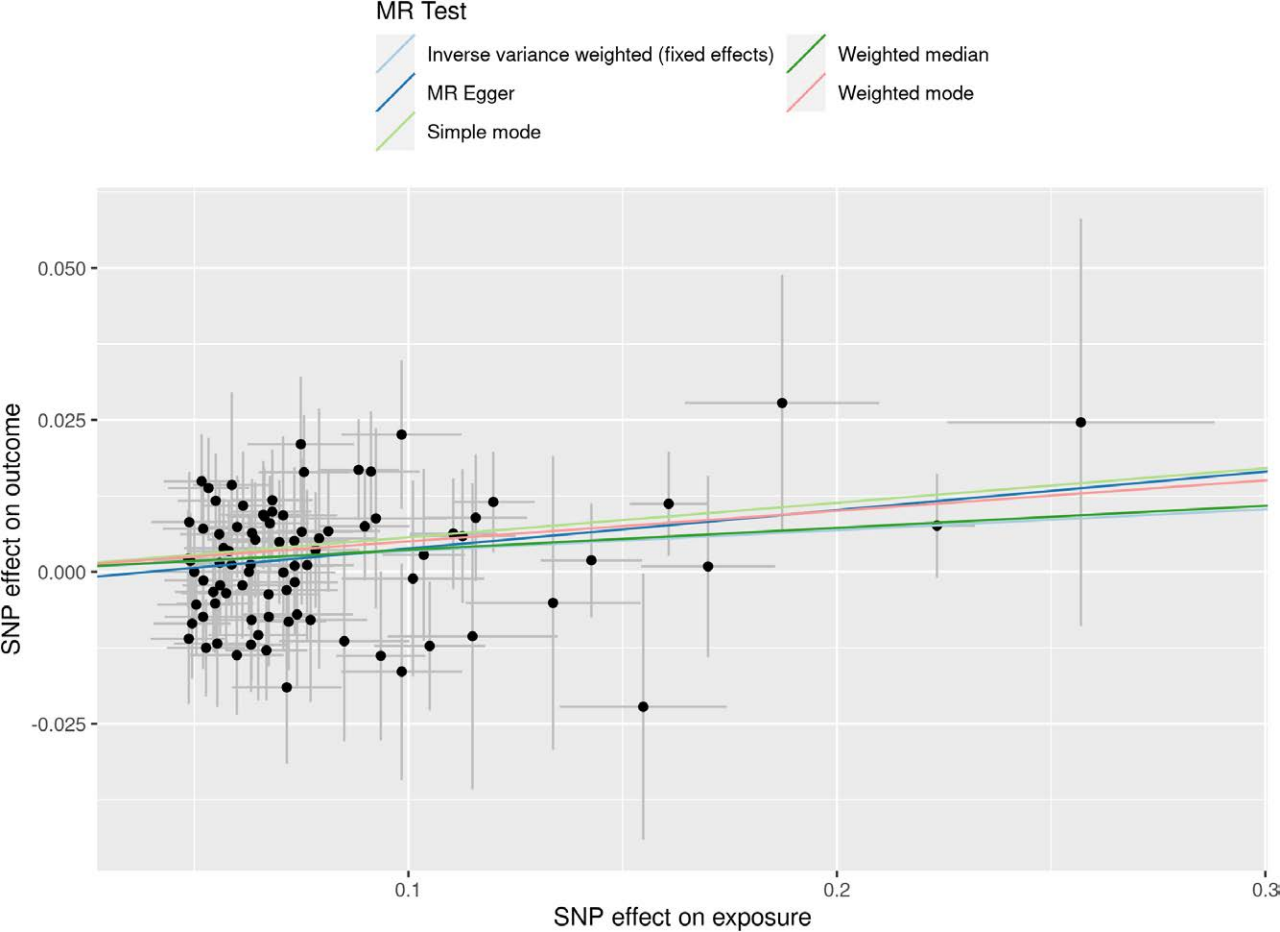
A scatter plot to show the SNPs effects on varicose veins and heart failure. Each black dot indicates a SNP, plotted by the estimate of SNP on varicose veins and the estimate of SNP on the risk of heart failure with standard error bars. The slopes of the straight lines indicate the magnitude of the causal association. IVW = inverse variance weighted, MR = Mendelian randomization, SNP = single nucleotide polymorphism.

**Figure 4.**
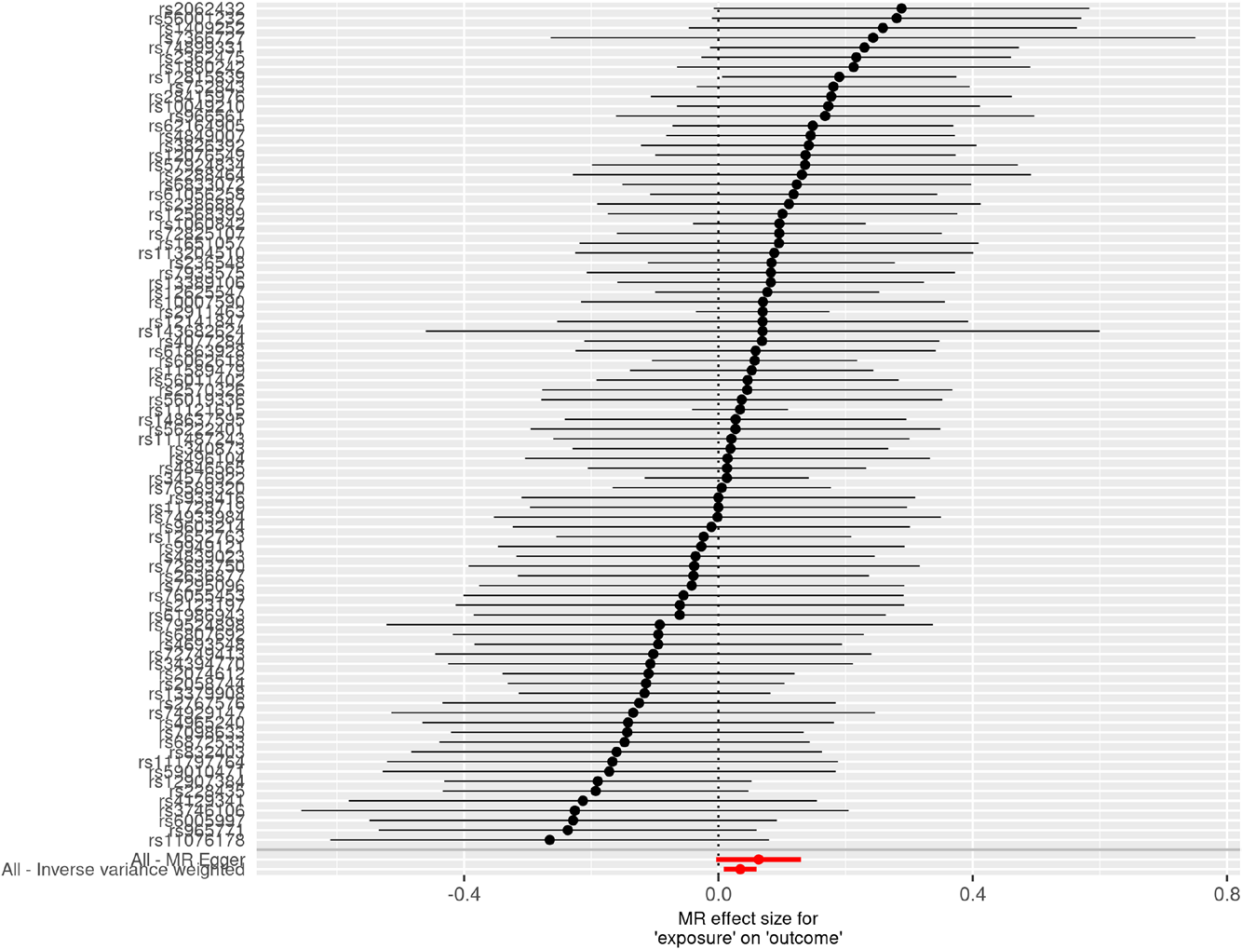
A forest plot to show the causal effect of each SNP on the risk of heart failure. The dot and bar indicate the causal estimate of varicose veins on risks of heart failure. MR = Mendelian randomization, SNP = single nucleotide polymorphism.

**Figure 5.**
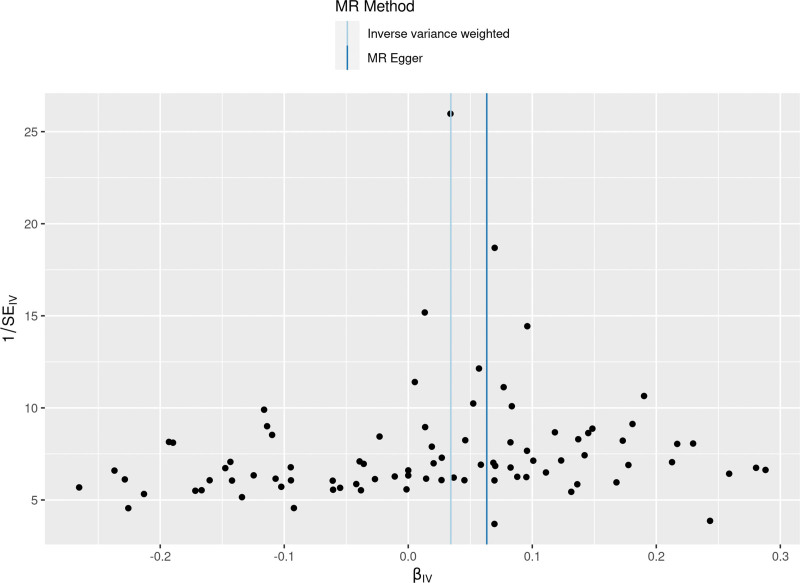
A funnel plots to show overall heterogeneity of MR estimates for the effect of varicose veins on the risk of heart failure. Each black dot indicates a SNP. MR = Mendelian randomization, SNP = single nucleotide polymorphism.

**Figure 6.**
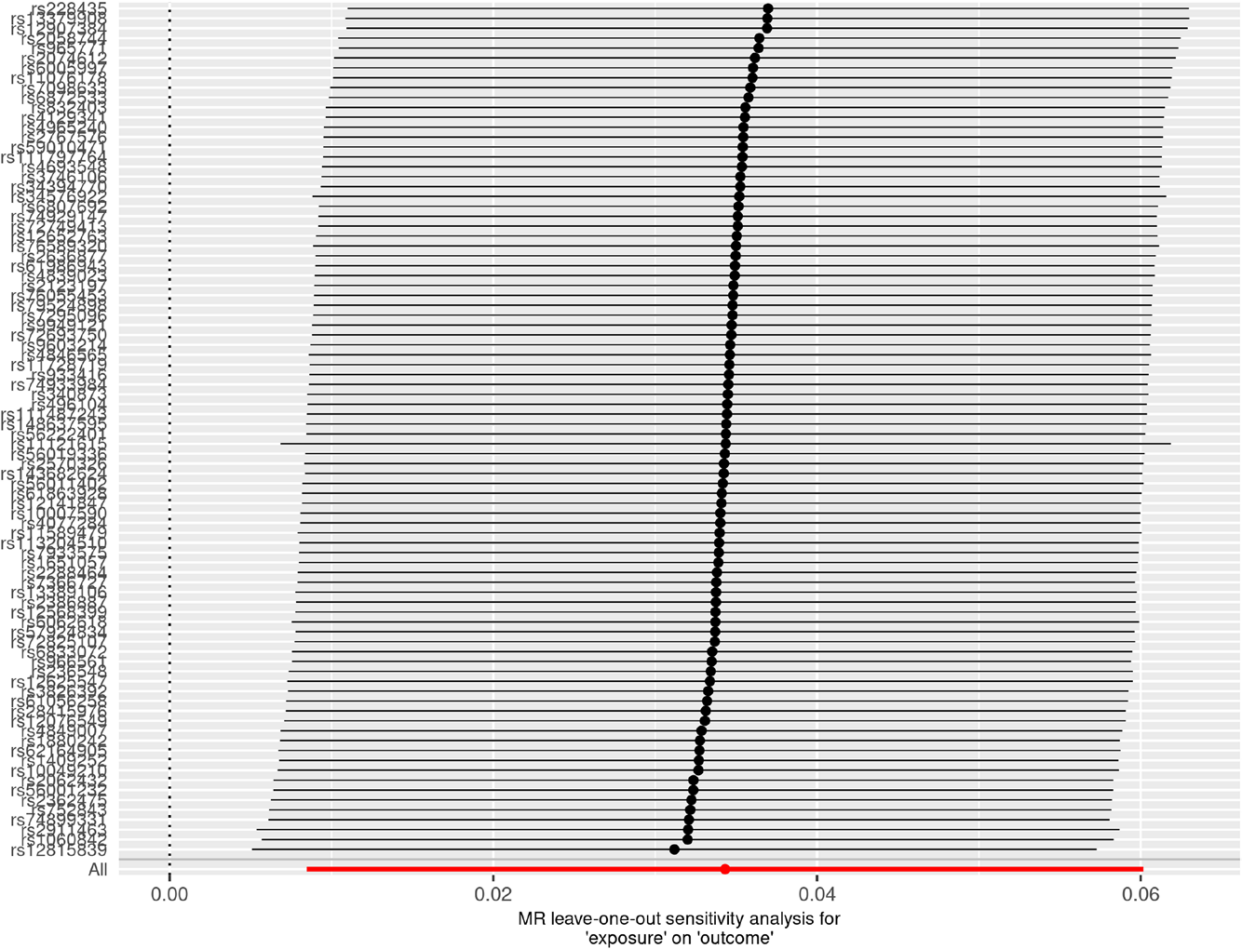
A leave-one-out plot to show causal effect of varicose veins on the risk of heart failure when leaving one SNP out. The dot and bar indicate the estimates and 95% confidence interval when the specific single nucleotide polymorphism is removed. MR = Mendelian randomization, SNP = single nucleotide polymorphism.

The results obtained through the inverse variance weighted method, as illustrated in Figure 2, demonstrated a significant association between genetically predicted varicose veins and an increased risk of HF (OR = 1.03; 95% CI: 1.01–1.06; *P* = .009). Additionally, the reverse MR analysis did not reveal any significant causal effect of HF on varicose veins. Notably, no significant heterogeneity of instrumental variables or horizontal pleiotropy was observed. Scatter plot and forest plot depicting the association between varicose veins and heart failure were presented in Figures 3 and 4, respectively, reflecting similar results. Furthermore, the funnel plot in Figure 5 also provided no evidence of horizontal pleiotropy. The leave-one-out sensitivity analysis, shown in Figure 6, indicated that the overall estimates were not disproportionately affected by any individual SNP.

To ensure the consistency of the findings across different datasets, the replicated MR analysis was conducted using UK biobank data. The results revealed a similar significant causal relationship between varicose veins and HF (Figures S1–S5, Supplemental Digital Content, http://links.lww.com/MD/M509, http://links.lww.com/MD/M531, http://links.lww.com/MD/M532, http://links.lww.com/MD/M533, http://links.lww.com/MD/M534).

The reverse MR analyses revealed no significant causal effect of genetic predisposition to HF on the risk of varicose veins (Table S5, Supplemental Digital Content, http://links.lww.com/MD/M508, Figures S6–S10, Supplemental Digital Content, http://links.lww.com/MD/M535, http://links.lww.com/MD/M536, http://links.lww.com/MD/M537, http://links.lww.com/MD/M538, http://links.lww.com/MD/M539).

## 4. Discussion

This study represents the first LDSC and MR study to comprehensively evaluate the genetic correlation and causal relationship between genetic predisposition to varicose veins and the risk of HF. Previous observational studies have suggested an elevated risk of HF in patients with varicose veins.^[8–13]^ Consistent with those findings, LDSC in our study showed that genetically predicted varicose veins were associated with increased susceptibility to heart failure, and MR analysis further a strong causal effect of varicose veins on the risk of heart failure in individuals of European descent. Furthermore, sensitivity analyses confirmed the positive association between these 2 conditions, and the causal effect of varicose veins on HF was found to be independent of confounding factors. In conclusion, our current MR study provides evidence of a causal, unidirectional relationship between varicose veins and the risk of HF, suggesting a heightened HF risk among individuals with varicose veins. Additionally, a reverse TSMR analysis of varicose veins and HF revealed that no significant causal relationship between HF and varicose veins. Although varicose veins were the potential risk of HF, the underlying biological mechanisms linking varicose veins and HF has not been well investigated previously. Wu et al^[9]^ and Jacob et al^[22]^ indicated that varicose veins-induced systemic inflammation may be associated with cardiovascular events.

To our knowledge, this study is the first to analyze the causal relationship between genetically predicted varicose veins and the risk of HF within the same study population using MR analysis. We obtained summary statistics from the latest and largest available GWAS data, ensuring robust instruments in the MR analysis. Horizontal pleiotropy was detected and excluded using MR-PRESSO and MR-Egger regression intercept term tests. As the alleles were randomly classified and fixed at conception in the TSMR, bias due to confusion and reverse causality was not observed in our study. Additionally, we observed consistent results across different datasets, which strengthened the robustness of our findings.

However, it is important to emphasize several limitations in our study. First, our study only included populations of European ancestry, potentially limiting its generalizability. Second, due to the lack of detailed clinic information, subgroup analyses were not possible. Third, despite efforts to minimize pleiotropy, it is unlikely to completely eliminate all instances of pleiotropy in MR analysis. Last, Although MR was able to avoid confounding bias compared to observational studies, the level of evidence was not as high as RCTS, therefore, further RCT experiments are needed to verify our results. There may still be unrecognized pathways and confounding factors between the exposure and outcome variables, potentially introducing biases into our results.

Nonetheless, our study provided evidence that patients with varicose veins should be monitored for HF risk. Therefore, the management and treatment of patients with varicose veins as a high-risk group has important potential value for the prevention of heart failure. Further research is necessary to explore the impact of varicose vein treatment on HF outcomes and investigate potential interventions targeting shared pathways between varicose veins and HF.

## 5. Conclusion

In this study, we presented compelling evidence of the causal relationship between varicose veins and the HF risk by MR analysis. Consequently, controlling the progression of varicose veins seemed to be an effective way to prevent HF. Further research is necessary to clarify the risk impact of varicose veins on HF, as well as specific pathways and mechanisms.

## Acknowledgments

We want to acknowledge the participants and investigators of FinnGen study and UK Biobank collaborators. The authors also appreciate the HFTSMR consortium for releasing the HF GWAS summary statistics.

## Author contributions

**Data curation:** Ping Guo.

**Funding acquisition:** Qin Fang.

**Supervision:** Qin Fang.

**Writing – original draft:** Ping Guo.

**Writing – review & editing:** Yan Wang.

## Supplementary Material

**Figure:**
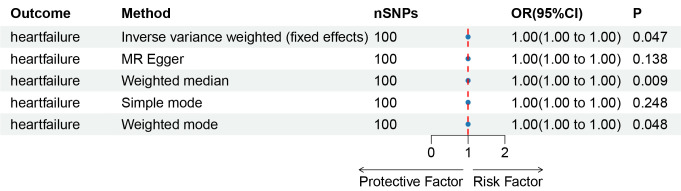


**Figure:**
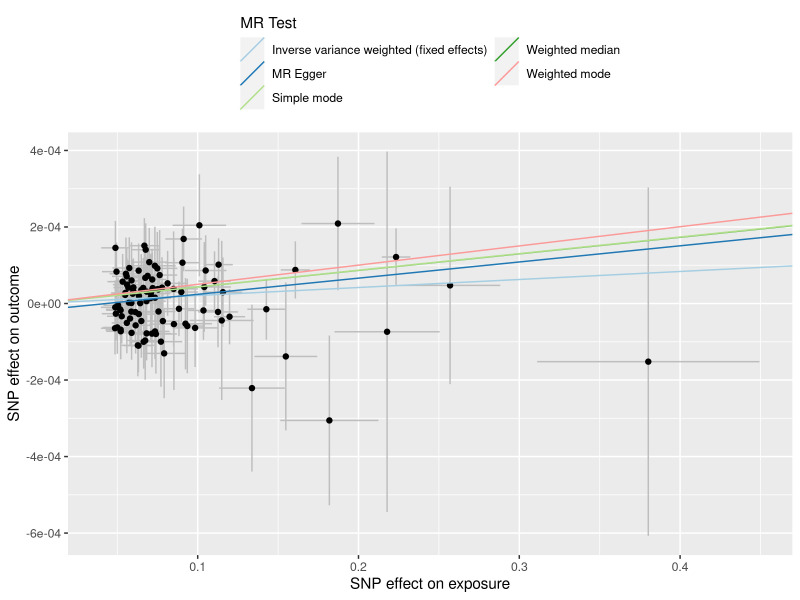


**Figure:**
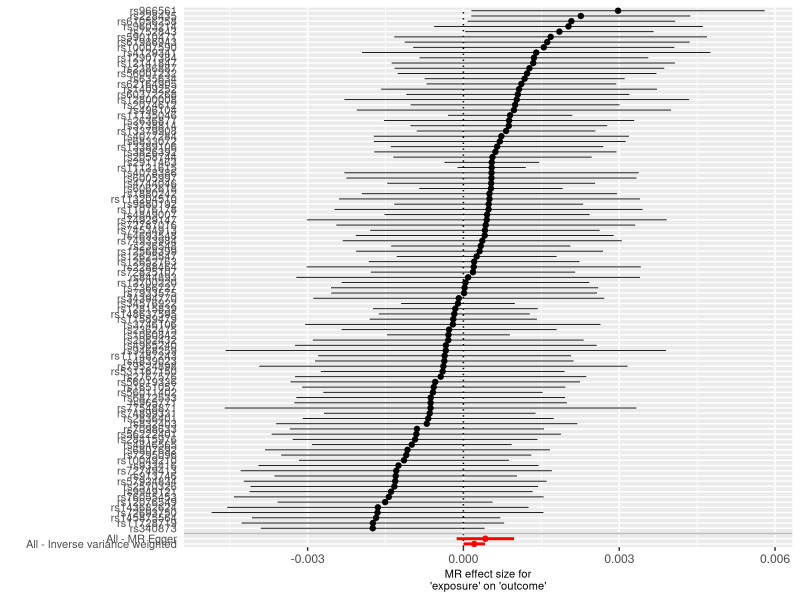


**Figure:**
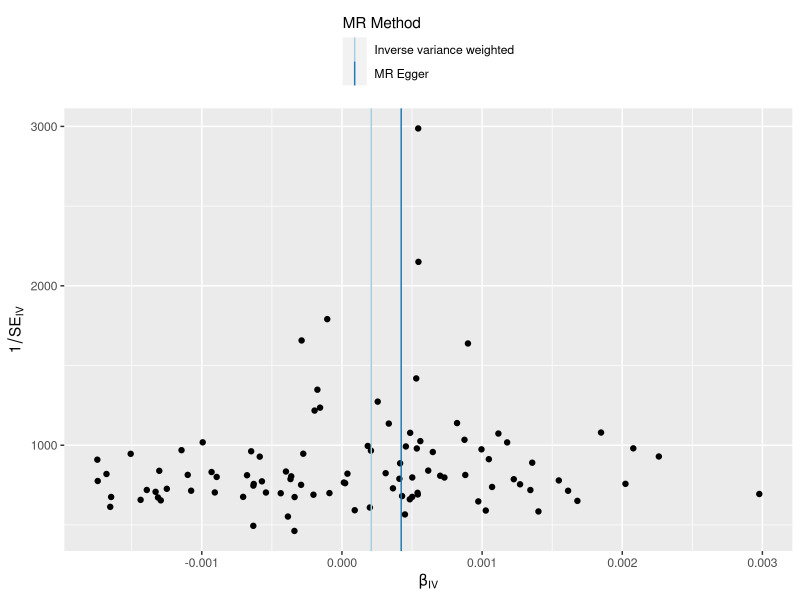


**Figure:**
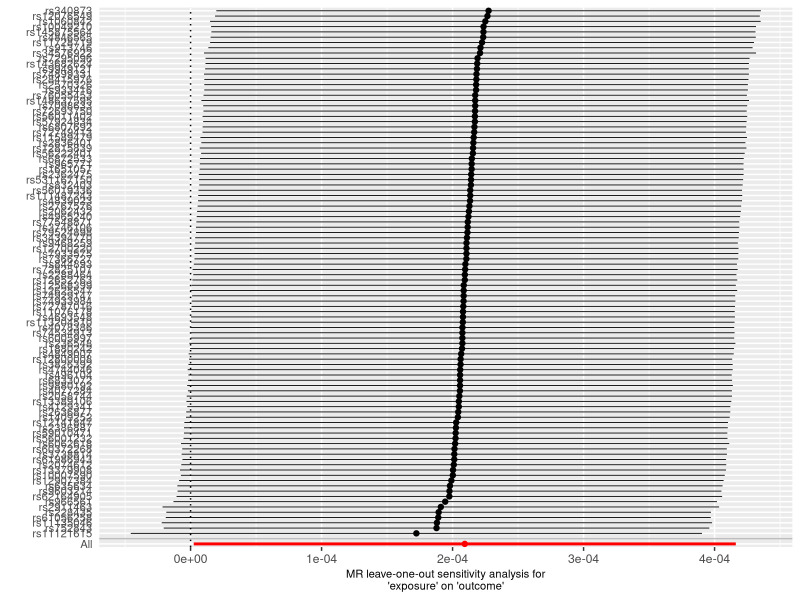


**Figure:**
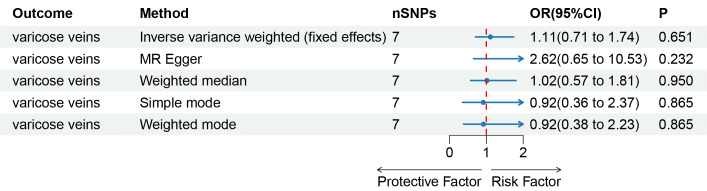


**Figure:**
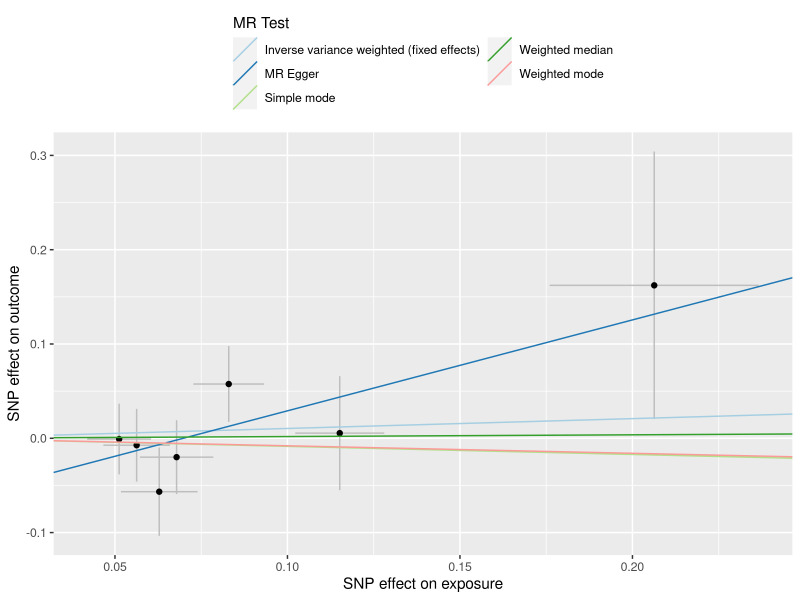


**Figure:**
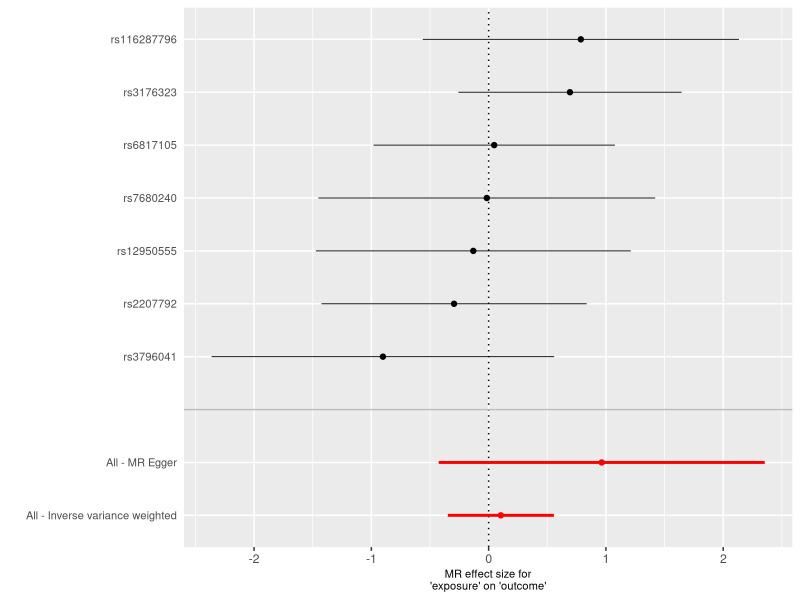


**Figure:**
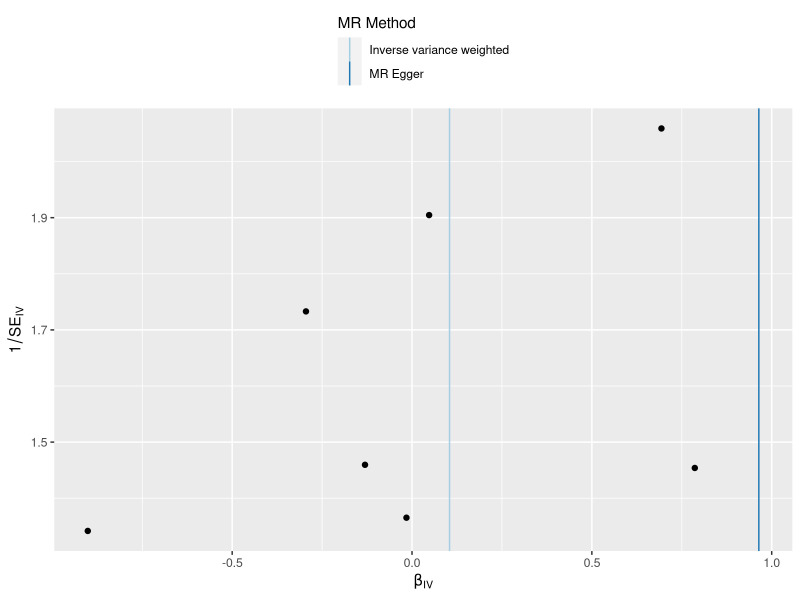


**Figure:**