# Supplementary material for: Associations between varicose veins and heart failure: A genetic correlation and mendelian randomization study
Source: Medicine (Baltimore). 2024 May 17;103(20):e38175. doi: 10.1097/MD.0000000000038175 (PMC11098184; doi:10.1097/MD.0000000000038175)
Supplement: Supplementary file 1 [file medi-103-e38175-s001.docx]

**Associations between varicose veins and heart failure: A genetic correlation and mendelian randomization study**

**Supplementary Table 1. Detailed information for the GWAS data of varicose veins and heart failure.**

| **Trait** | **GWAS ID** | **Ancestry** | **Sample Size** | **Case/ control** | **PMID** |
| --- | --- | --- | --- | --- | --- |
| Varicose veins | finngen_R10_I9_VARICVE | European | 388,830 | 31,719/357,111 | NA |
| heart failure | ukb_heartfailure_male_female | European | NA | NA | NA |
