# Supplementary material for: Associations between varicose veins and heart failure: A genetic correlation and mendelian randomization study
Source: Medicine (Baltimore). 2024 May 17;103(20):e38175. doi: 10.1097/MD.0000000000038175 (PMC11098184; doi:10.1097/MD.0000000000038175)
Supplement: Supplementary file 2 [file medi-103-e38175-s002.docx]

**Associations between varicose veins and heart failure: A genetic correlation and mendelian randomization study**

| **Trait** | **GWAS ID** | **Ancestry** | **Sample Size** | **Case/ control** | **PMID** |
| --- | --- | --- | --- | --- | --- |
| heart failure | finngen_R10_I9_HEARTFAIL_ALLCAUSE | European | 411,056 | 29,218/381,838 | NA |
| Varicose veins | Categorical-20002-both_sexes-1494 | European | 420,473 | 1,510/418,963 | NA |

**Supplementary Table 2. Detailed information for the GWAS data of heart failure and varicose veins.**
