# Supplementary material for: Associations between varicose veins and heart failure: A genetic correlation and mendelian randomization study
Source: Medicine (Baltimore). 2024 May 17;103(20):e38175. doi: 10.1097/MD.0000000000038175 (PMC11098184; doi:10.1097/MD.0000000000038175)
Supplement: Supplementary file 3 [file medi-103-e38175-s003.docx]

**Associations between varicose veins and heart failure: A genetic correlation and mendelian randomization study**

**Supplementary Table 3. The genetic correlations between varicose veins and heart failure.**

| **Trait** | **mean_**  **chisq** | **lambda_gc** | **intercept** | **intercept_se** | **ratio** | **ratio_se** | **h2_observed** | **h2_observed_se** | **h2_Z** | **h2_p** |
| --- | --- | --- | --- | --- | --- | --- | --- | --- | --- | --- |
| Varicose veins | 1.656550 | 1.486445 | 1.267639 | 0.033371507 | 0.4076441 | 0.05082859 | 0.051120615 | 0.0052700341 | 9.700244 | 3.007794e-22 |
| heart failure | 1.159402 | 1.128973 | 1.020264 | 0.007311609 | 0.1271236 | 0.04586906 | 0.006954643 | 0.006112174 | 11.378346 | 5.360674e-30 |
