# Supplementary material for: Associations between varicose veins and heart failure: A genetic correlation and mendelian randomization study
Source: Medicine (Baltimore). 2024 May 17;103(20):e38175. doi: 10.1097/MD.0000000000038175 (PMC11098184; doi:10.1097/MD.0000000000038175)
Supplement: Supplementary file 4 [file medi-103-e38175-s004.docx]

**Associations between varicose veins and heart failure: A genetic correlation and mendelian randomization study**

**Supplementary Table 4. Single nucleotide polymorphisms used as instrumental variables in the Mendelian randomization analyses of varicose veins.**

| **SNP** | **Effect_ allele. exposure** | **Other_ allele. exposure** | **se. exposure** | **beta. exposure** | **pos. exposure** |
| --- | --- | --- | --- | --- | --- |
| rs11121615 | T | C | 0.00888 | -0.22336 | 10765520 |
| rs2911463 | A | G | 0.009074 | -0.16074 | 88769137 |
| rs11135046 | T | G | 0.008847 | -0.11299 | 1.59E+08 |
| rs28558138 | C | G | 0.008893 | -0.11331 | 26816458 |
| rs1060842 | T | G | 0.009661 | 0.119808 | 68174880 |
| rs34576922 | G | C | 0.011825 | 0.142695 | 1.28E+08 |
| rs6062618 | G | T | 0.010134 | -0.11046 | 64051176 |
| rs76589320 | T | C | 0.015611 | -0.16993 | 88470155 |
| rs148637595 | G | C | 0.00964 | 0.103577 | 47819603 |
| rs12625547 | G | T | 0.011942 | -0.1157 | 51538108 |
| rs12815839 | C | T | 0.009416 | 0.088361 | 50778350 |
| rs11589479 | A | G | 0.012384 | 0.112623 | 1.55E+08 |
| rs6872533 | A | G | 0.010407 | -0.09354 | 1.31E+08 |
| rs236548 | A | G | 0.010425 | -0.08981 | 70235111 |
| rs635634 | C | T | 0.010774 | -0.09052 | 1.33E+08 |
| rs4744046 | G | T | 0.009334 | 0.077835 | 91450712 |
| rs62164905 | G | A | 0.022681 | -0.18723 | 1.18E+08 |
| rs72825107 | G | A | 0.031187 | -0.25695 | 39297558 |
| rs2058744 | A | G | 0.00877 | -0.07204 | 72044167 |
| rs3738814 | G | A | 0.008774 | -0.07174 | 17005181 |
| rs1372809 | G | C | 0.008763 | 0.071445 | 10222558 |
| rs9880192 | C | G | 0.009306 | -0.07548 | 1.29E+08 |
| rs13379908 | C | T | 0.01294 | -0.10496 | 95633073 |
| rs4846565 | A | G | 0.009059 | 0.073442 | 2.2E+08 |
| rs752843 | T | C | 0.011396 | 0.091254 | 88702696 |
| rs7098633 | C | T | 0.019521 | -0.1548 | 62054438 |
| rs2074612 | C | T | 0.008724 | -0.06741 | 1.4E+08 |
| rs56011402 | G | A | 0.010276 | 0.07832 | 1.72E+08 |
| rs12076549 | C | A | 0.008773 | -0.06638 | 91736142 |
| rs4849007 | A | C | 0.009147 | -0.06821 | 1.12E+08 |
| rs61056258 | A | G | 0.009133 | -0.06766 | 37526483 |
| rs60372268 | G | A | 0.009317 | 0.068857 | 46870390 |
| rs12652763 | A | G | 0.010006 | 0.073469 | 1.73E+08 |
| rs10049210 | C | T | 0.009353 | -0.06823 | 1.57E+08 |
| rs12907384 | C | T | 0.008765 | -0.06326 | 85732769 |
| rs340873 | A | G | 0.008779 | -0.06315 | 2.14E+08 |
| rs2362475 | C | A | 0.010558 | -0.07562 | 16329024 |
| rs74899331 | A | C | 0.014014 | -0.09839 | 89200688 |
| rs496104 | A | G | 0.010896 | 0.076344 | 1.71E+08 |
| rs228435 | T | C | 0.009578 | 0.066848 | 1.35E+08 |
| rs111797764 | T | C | 0.014114 | 0.098423 | 12463340 |
| rs2471980 | C | G | 0.008844 | -0.05984 | 31833091 |
| rs6833072 | C | T | 0.00889 | -0.06002 | 1.19E+08 |
| rs13389106 | G | T | 0.012071 | -0.0813 | 30257884 |
| rs3826392 | T | G | 0.009866 | 0.066113 | 12019587 |
| rs12568399 | C | T | 0.009499 | -0.06349 | 2.22E+08 |
| rs9468259 | G | A | 0.032689 | 0.217786 | 27933283 |
| rs2836401 | C | T | 0.008806 | 0.057924 | 38443653 |
| rs72693750 | C | T | 0.020434 | 0.133747 | 1.66E+08 |
| rs913746 | C | A | 0.009001 | 0.058881 | 1.1E+08 |
| rs12700220 | C | T | 0.008773 | 0.057242 | 20575260 |
| rs111487243 | T | C | 0.009021 | -0.05873 | 86557196 |
| rs7366727 | T | C | 0.00905 | 0.058764 | 9411592 |
| rs28415976 | G | A | 0.009464 | 0.061403 | 50291791 |
| rs2636877 | C | T | 0.008816 | -0.05605 | 1.14E+08 |
| rs74534913 | T | C | 0.016517 | -0.10409 | 1.3E+08 |
| rs1880242 | T | G | 0.008865 | -0.05501 | 22719988 |
| rs11728719 | C | A | 0.010118 | -0.06277 | 1.86E+08 |
| rs113204510 | A | G | 0.012104 | 0.075094 | 73387015 |
| rs4839023 | C | G | 0.00993 | 0.061255 | 1.15E+08 |
| rs4077284 | G | A | 0.009245 | -0.05685 | 73936050 |
| rs12800008 | A | C | 0.009601 | -0.05885 | 1.28E+08 |
| rs10007590 | G | A | 0.011414 | 0.069873 | 88995984 |
| rs2386887 | C | A | 0.00914 | 0.055842 | 4254565 |
| rs4693548 | A | G | 0.009007 | 0.054884 | 83036531 |
| rs2123197 | A | G | 0.008958 | 0.054447 | 47127589 |
| rs9603214 | C | T | 0.016653 | 0.101046 | 37490980 |
| rs56001232 | T | C | 0.012439 | -0.07488 | 1.15E+08 |
| rs2767576 | C | T | 0.010558 | 0.063379 | 1.57E+08 |
| rs7295096 | G | A | 0.01194 | 0.071559 | 27644972 |
| rs61863928 | T | G | 0.009704 | -0.05808 | 62689789 |
| rs145875564 | A | G | 0.030529 | -0.18188 | 79394009 |
| rs56222401 | G | A | 0.009409 | -0.05593 | 49278931 |
| rs143682624 | A | G | 0.013378 | 0.079144 | 25288262 |
| rs2062432 | A | G | 0.008824 | 0.051722 | 1.23E+08 |
| rs72749413 | T | C | 0.013248 | -0.07715 | 1.98E+08 |
| rs74933984 | A | G | 0.012168 | -0.07081 | 42894710 |
| rs10152793 | C | G | 0.009005 | 0.052267 | 22889021 |
| rs548064470 | A | C | 0.028676 | -0.16619 | 1.46E+08 |
| rs4965240 | A | G | 0.008998 | 0.052069 | 99460546 |
| rs79524898 | A | G | 0.019865 | -0.11495 | 64168374 |
| rs76055453 | C | T | 0.011656 | 0.067351 | 70036623 |
| rs965771 | G | A | 0.009151 | -0.05277 | 98116592 |
| rs7933575 | G | A | 0.011179 | -0.06424 | 1567902 |
| rs9949121 | T | G | 0.009092 | -0.05214 | 44726919 |
| rs844893 | T | C | 0.011956 | -0.06814 | 23130619 |
| rs6807692 | T | C | 0.013053 | -0.07401 | 61649920 |
| rs12141847 | G | A | 0.01299 | -0.07336 | 81614025 |
| rs1409252 | G | A | 0.009454 | 0.053331 | 75128519 |
| rs2288464 | A | C | 0.012584 | 0.070751 | 17306443 |
| rs56019336 | G | A | 0.00876 | -0.0491 | 2.02E+08 |
| rs832403 | T | G | 0.011609 | -0.06494 | 56970165 |
| rs74929147 | A | G | 0.0152 | -0.08502 | 18302251 |
| rs1651057 | G | A | 0.016521 | -0.0924 | 31673324 |
| rs11076178 | T | C | 0.012803 | 0.071599 | 57112490 |
| rs77548871 | C | G | 0.015325 | 0.085213 | 1.55E+08 |
| rs933416 | A | C | 0.009012 | -0.05002 | 3092204 |
| rs72787016 | C | T | 0.010746 | -0.0596 | 1.31E+08 |
| rs34394770 | C | T | 0.009112 | 0.050476 | 39759232 |
| rs4078386 | G | C | 0.011731 | 0.064908 | 19181556 |
| rs2570326 | T | C | 0.008803 | 0.04858 | 34224902 |
| rs57924834 | A | G | 0.009449 | -0.05209 | 1.01E+08 |
| rs966561 | G | A | 0.008869 | 0.04884 | 6797870 |
| rs2576380 | G | C | 0.009094 | 0.050013 | 1.01E+08 |
| rs531167150 | T | A | 0.069143 | 0.380217 | 70051406 |
| rs59010471 | G | A | 0.009027 | 0.049509 | 2.18E+08 |
| rs6005997 | C | T | 0.010933 | 0.059944 | 29122228 |
| rs4129341 | G | T | 0.010104 | 0.055378 | 9644867 |
| rs61986943 | T | C | 0.010502 | -0.05745 | 68506382 |
| rs3746106 | A | C | 0.00893 | -0.04873 | 1250110 |

SE: standard error; SNP: single-nucleotide polymorphisms.
