# Supplementary material for: Associations between varicose veins and heart failure: A genetic correlation and mendelian randomization study
Source: Medicine (Baltimore). 2024 May 17;103(20):e38175. doi: 10.1097/MD.0000000000038175 (PMC11098184; doi:10.1097/MD.0000000000038175)
Supplement: Supplementary file 5 [file medi-103-e38175-s010.docx]

**Associations between varicose veins and heart failure: A genetic correlation and mendelian randomization study**

**Supplementary Table 5. Single nucleotide polymorphisms used as instrumental variables in the Mendelian randomization analyses of HF.**

| **SNP** | **Effect_ allele. exposure** | **Other_ allele. exposure** | **se. exposure** | **beta. exposure** | **pos. exposure** | **pval. exposure** |
| --- | --- | --- | --- | --- | --- | --- |
| rs6817105 | C | T | 0.012904 | 0.115153 | 1.11E+08 | 4.50E-19 |
| rs3176323 | C | T | 0.010258 | -0.08298 | 36679072 | 6.00E-16 |
| rs116287796 | T | C | 0.030314 | 0.206311 | 50991770 | 1.00E-11 |
| rs2207792 | A | G | 0.010641 | -0.06786 | 61429037 | 1.80E-10 |
| rs12950555 | G | C | 0.009649 | -0.05628 | 2253616 | 5.45E-09 |
| rs3796041 | A | C | 0.011099 | -0.06283 | 36990479 | 1.51E-08 |
| rs7680240 | C | A | 0.009266 | -0.05121 | 1.11E+08 | 3.26E-08 |
